# Examination of *SOX2* in variable ocular conditions identifies a recurrent deletion in microphthalmia and lack of mutations in other phenotypes

**Published:** 2010-04-28

**Authors:** Linda M. Reis, Rebecca C. Tyler, Adele Schneider, Tanya Bardakjian, Elena V. Semina

**Affiliations:** 1 Department of Pediatrics and Children’s Research Institute at the Medical College of Wisconsin and Children’s Hospital of Wisconsin, Milwaukee, WI; 2 Albert Einstein Medical Center, Department of Pediatrics Division of Genetics, Philadelphia, PA; 3 Department of Cell Biology, Neurobiology and Anatomy at the Medical College of Wisconsin, Milwaukee, WI

## Abstract

**Purpose:**

The role of SRY-Box 2 (*SOX2*) in anophthalmia/microphthalmia (A/M) is well known, with 10%–20% of A/M explained by mutations in *SOX2*. SOX2 plays roles in the development of both the posterior and anterior segment structures of the eye and relies on interactions with tissue-specific partner proteins to execute its function, raising the possibility that *SOX2* mutations may result in varying ocular phenotypes. Recent data has identified a missense mutation in *SOX2* in an extended pedigree with phenotypes as varied as A/M, isolated iris hypoplasia, iris and chorioretinal coloboma, pupil defects, and hypermetropia, suggesting a broader phenotypic spectrum associated with *SOX2* mutations.

**Methods:**

Screening of *SOX2* was completed in 89 patients with a variety of ocular anomalies, including 28 with A/M and 61 with normal eye size and anterior segment dysgenesis (28), cataract (14), isolated coloboma (5), or other eye disorders (14).

**Results:**

The recurrent de novo frameshift mutation c.70del20 was identified in one patient with microphthalmia and syndromic anomalies consistent with *SOX2* anophthalmia syndrome; the mutation frequency in our A/M population (4%) was lower than previously reported; it is likely that extensive utilization of clinical *SOX2* testing has led to a bias toward *SOX2*-negative A/M cases in our research cohort. No disease-causing mutations were identified in patients with non-microphthalmia phenotypes.

**Conclusions:**

The recurrent c.70del20 mutation accounts for 21% of all independent *SOX2* mutations reported to date. Due to the increased use of clinical *SOX2* testing, the frequency of *SOX2* mutations identified in research A/M populations will likely continue to decrease. Mutations in *SOX2* do not appear to be a common cause of ocular defects other than anophthalmia/microphthalmia.

## Introduction

SOX2 is a High Mobility Group (HMG) DNA binding domain-containing transcription factor with a well established role as a common cause of dominant anophthalmia/microphthalmia (A/M). *SOX2/Sox2* expression begins early in eye development and is critical for the formation of different ocular tissues [1-4]. In mice, *Sox2* is expressed within the optic vesicle and stalk, the developing neural retina, the placodal area of the surface ectoderm, and throughout lens development starting with the lens placode and continuing through differentiating lens fibers [1,2,4]. Studies of human fetal tissues are consistent with the mouse data; *SOX2* expression is seen in the developing neural retina and optic stalk as well as during all stages of lens development [3].

SOX proteins typically contain three functional domains: the High Mobility Group (HMG) domain that is responsible for DNA binding, an activation or repression domain, and a partner-factor interaction domain. SOX2, like other SOX proteins, requires interaction with partner factors to successfully regulate target genes, likely due to low-affinity DNA-binding by SOX proteins alone [5]. Partner factors bind to the regulatory sequence near the site of SOX binding and also interact with the SOX protein, thus forming a ternary complex. This complex stabilizes the interaction between the SOX protein and DNA and allows specific regulation of diverse target genes [5]. For example, SOX2 has been shown to partner with δ-crystallin enhancer factor 3 (δEF3) as well as paired box gene 6 (PAX6) to activate δ-crystallin expression during lens formation [6,7] and with octomer-binding transcription factor 3/4 (Oct3/4) to regulate fibroblast growth factor 4 (*FGF4*) or undifferentiated embryonic cell transcription factor 1 (*UTF1*) target genes in embryonic stem cells [5]. Thus, depending on the affected site/domain, it is possible that some *SOX2* mutations may affect certain protein interactions more than others and therefore result in variable and tissue-specific phenotypes.

Mutations in *SOX2* account for 10%–20% of A/M [8-11]. Individuals with *SOX2* mutations often have associated systemic anomalies, termed *SOX2* anophthalmia syndrome, consisting of ocular, brain, pituitary, genitourinary, and gastresophageal anomalies, although eye defects can be isolated as well [8-11]. The anophthalmia/microphthalmia phenotype in patients with *SOX2* mutations can be bilateral, unilateral, or, occasionally, absent; 14 of the 71 individuals reviewed previously [11] or subsequently reported [12] do not have anophthalmia/microphthalmia of either eye. Severe *SOX2* mutations (whole gene deletion/nonsense) which most likely produce complete loss-of-function alleles almost uniformly result in anophthalmia/microphthalmia (47 out of 50 cases [11]), suggesting that these mutations result in a major disruption of eye development, while missense mutations are more likely to be associated with non-microphthalmia phenotypes (11 of 21 cases [11,12]).

Several families with missense mutations and non-microphthalmia phenotypes have been previously reported including two probands with optic nerve hypoplasia and their unaffected fathers [13], a father and son with iris and chorioretinal uveal colobomas [14], and, more recently, a large four-generation pedigree with diverse phenotypes, including microphthalmia in some individuals [12]. The latest report describes a mutation in a four-generation pedigree that results in the substitution of a highly conserved amino acid within the partner-factor interaction region of the SOX2 protein and is the first mutation reported in this domain. While the proband was affected with bilateral anophthalmia, the other eight family members carrying the mutation were affected with milder ocular phenotypes. Five of the affected individuals did not have anophthalmia or microphthalmia. The proband’s mother had bilateral iris and chorioretinal colobomata, the maternal aunt was found to have a small inferior retinal tuft in one eye along with hypermetropia and astigmatism, and the proband’s brother was affected with hypermetropia only. Finally, the maternal grandmother was affected with iris hypoplasia and hypermetropia while a maternal great-aunt had microcornea only. In addition to the two cases above with isolated anterior segment defects (iris hypoplasia and microcornea), two individuals demonstrated anterior segment defects (posterior embryotoxon and an iris pupillary defect) in the presence of microphthalmia, further suggesting that *SOX2* plays a role in anterior segment development. Several family members who did not carry the mutation had normal eye examinations [12].

As discussed above, the variability of phenotypes associated with, in particular, missense changes in *SOX2* may be explained by the fact that these mutations result in alteration of its interactions with tissue-specific protein partners rather than a complete loss-of-function [5,12-14]. In addition to this, the genetic makeup of these partner factors, other ocular development genes, or yet unknown factors are likely to play role in the phenotypic expression of *SOX2* mutations. Since expression during lens development represents an important site of SOX2 activity, it is possible that some mutations may lead to phenotypes associated with specific loss of SOX2-related activity in the lens, such as cataracts and anterior segment defects.

Mutations in *SOX2* may result in a broader phenotypic spectrum consistent with its role in both posterior and anterior segment development and dependent on the affected partner interaction domain. The role of *SOX2* in A/M has been well characterized while its potential involvement in other eye conditions has not yet been investigated. To further explore the role of *SOX2* in variable ocular diseases, we undertook screening of this gene in our population of patients with a variety of ocular defects.

## Methods

This human study was approved by the Institutional Review Boards of Children's Hospital of Wisconsin and Albert Einstein Healthcare Network. Cases were identified and clinical data collected through the Genetic Studies of Human Developmental Disorders at the Medical College of Wisconsin, Milwaukee, WI or the A/M Clinical Registry at the Albert Einstein Medical Center, Philadelphia, PA. Genomic DNA was extracted using the Autopure LS system (Qiagen, Valencia, CA) from blood or buccal samples and the *SOX2* coding region was amplified using primers: set 1 forward, 5'-AGT CCC GGC CGG GCC GAG-3', and set 1 reverse, 5'-GGT AGC CCA GCT GGT CCT G-3', and set 2 forward, 5'-CAA GAC GCT CAT GAA GAA GG-3', and set 2 reverse, 5'-TAC TCT CCT CTT TTG CAC CC-3'. PCR products were sequenced in both directions using Big Dye Terminator v3.1 (Applied Biosystems, Foster City, CA) with 3730XL DNA Analyzer (Applied Biosystems, Foster City, CA). Chromatograms were examined manually and using Mutation Surveyor software (SoftGenetics, State Collge, PA). All initially identified changes were confirmed by additional independent PCR and sequencing experiments.

The screening included 89 patients with a variety of ocular disorders (Table 1). Twenty-eight patients were affected with anophthalmia/microphthalmia (17 bilateral, 17 syndromic). An additional 28 patients had anterior segment dysgenesis (22 bilateral, 20 syndromic); eight of these patients were specifically noted to have iris hypoplasia (two with full Axenfeld-Rieger syndrome), four were noted to have pupil anomalies, 11 had Peters’ anomaly (one with iris coloboma in addition), and the remaining five had other anterior segment defects. There were five patients with isolated coloboma (three were iris and chorioretinal). Finally, six patients had isolated high myopia, four patients had glaucoma with no other ocular anomalies (three congenital, one adult-onset), 14 had cataract with no other ocular anomalies (11 congenital, two juvenile, and one adult-onset), and the remaining four had other eye defects (including microspherophakia, Septo-optic dysplasia, and Morning Glory disc anomaly).

**Table 1:** Summary of ocular conditions and *SOX2* mutations reported in this study.

**Ocular condition**	**Number of cases**	**Bilateral**	**Syndromic**	**Number of mutations**
Anophthalmia	8	6/8	6/8	0
Microphthalmia	20	11/20	11/20	1
**A/M total**	**28**	**17/28**	**17/28**	**1/28**
Anterior segment dysgenesis	28	22/28	20/28	0
Cataract	14	14/14	1/14	0
High myopia	6	6/6	0/6	0
Coloboma (isolated)	5	3/5	0/5	0
Glaucoma	4	4/4	2/4	0
Other eye defect	4	4/4	4/4	0
**Non-A/M total**	**61**	**53/61**	**27/61**	**0/61**
**Total number**	**89**	**70/89**	**44/89**	**1/89**

Paired homeodomain transcription factor 2 (*PITX2*) was previously screened in 26 of the 28 patients with anterior segment dysgenesis with no mutations identified. Forkhead box E3 (*FOXE3*) was previously screened in 20 of the 28 patients with anterior segment dysgenesis (including 9 of 11 with Peters’ anomaly) with no mutations identified. Two patients with mutations in *SOX2* identified via clinical testing were known to decline enrollment in this research study during this period of patient recruitment.

## Results and Discussion

**Figure 1:**
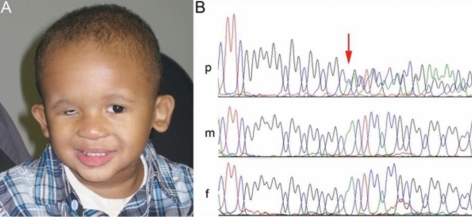
Identification of a c.70del20 mutation in a patient with unilateral microphthalmia. **A**: Photograph of Patient 1 with *SOX2* anophthalmia syndrome. Note right microphthalmia (prosthesis in place) and prominent ears. **B**: Sequence fragments showing the c.70del20 region in the patient (p), his mother (m) and his father (f). The position of the deletion is indicated with a red arrow. Note normal *SOX2* sequence in the patient’s parents consistent with their unaffected status.

Screening of these 89 patients identified a disease-causing mutation in one patient. The recurrent frameshift mutation in *SOX2*, c.70del20, was identified in Patient 1 of African American decent who was affected with microphthalmia and optic nerve hypoplasia in the right eye but had a normal left eye; in addition to ocular defects, the patient displayed micropenis, cryptorchidism, prostatic utricle, pancreatic deficiency, low-set prominent ears, umbilical hernia, feeding disorder, and global developmental delay, consistent with *SOX2* anophthalmia syndrome (Figure 1A,B). This mutation is predicted to result in a frameshift and truncation of normal SOX2 protein at amino acid 23 (7% of its total length), before the HMG DNA-binding domain. The parents of Patient 1 are unaffected and were found to carry normal *SOX2* alleles consistent with the de novo phenotype observed in their child (Figure 1B). The recurrent c.70del20 mutation has now been reported in eleven patients from ten unrelated pedigrees, typically resulting in a severe phenotype characterized by bilateral anophthalmia/microphthalmia and systemic anomalies (Table 2). The family reported in this study represents only the second case of unilateral eye defects associated with the c.70del20 mutation; the previously described pedigree includes a patient with unilateral ocular phenotype and a sibling with normal eyes bilaterally [15]. Also, to the best of our knowledge, this is the first reported occurrence of the c.70del20 mutation in an African American patient, but several previous reports did not include race/ethnicity data. The c.70del20 mutation appears to be the major recurrent mutation in *SOX2*; one other mutation, c.529C>T, has been noted in three unrelated families [8,13] while the remaining 34 reported mutations are unique [11]. Two similar deletions, a 17-base pair deletion at the same nucleotide position, and a 23-base pair deletion in the codon before, have each been reported in one family [9,16]. Slipped-strand mispairing has been implicated as the likely mechanism of this recurrent mutation due to the presence of a GGCGGC repeat sequence flanking the region [17]. These 17 to 23-base pair deletions account for 25% of all *SOX2* mutations in probands (12/48) with the c.70del20 mutation contributing 21% (10/48). This warrants special attention to this region in A/M patients being evaluated for *SOX2* mutations.

**Table 2:** Clinical findings in patients with the recurrent c.70del20 mutation in *SOX2*.

**Reported case**	**1**	**2**	**3 (sibling)**	**4**	**5**	**6**	**7**	**8**	**9**	**10**	**11**
Right Eye	AN	NOR	NOR	MI	AN	AN	AN	MI	AN	AN	MI, ONH
Left Eye	AN	AN	NOR	AN	ASD, CA, COL, GL	AN	AN	MI	AN	AN	NOR
Other Anomalies	Yes	Yes	Yes	Yes	Yes	No	Yes	Yes	Yes	Yes	Yes
Reference	[17]	[15]	[15]	[12]	[10]	[10]	[3]	[11]	[11]	[11]	This study

The reported cases are numbered based upon date of publication. In the table AN indicates Anophthalmia; MI indicates Microphthalmia; ASD indicates Anterior segment dysgenesis; CA indicates Cataract; COL indicates Coloboma; GL indicates Glaucoma; NOR indicates Normal; and ONH indicates Optic nerve hypoplasia.

The frequency of *SOX2* mutations within our population of patients with anophthalmia/ microphthalmia (1/28=4%) was much lower than has been previously reported [8-11], likely due to increased clinical testing of *SOX2* in affected patients before enrollment in a research study. If the two patients known to have a clinically identified mutation in *SOX2* are included, the frequency of *SOX2* mutations increases to 10% (3/30), which is consistent with previous reports. Given the well known role of *SOX2* in anophthalmia/microphthalmia, the easily recognizable associated features, and the availability of clinical sequencing, it seems likely that many physicians may order clinical testing before referring a family to a research study. Thus, the frequency of *SOX2* mutations observed in current and future research populations will likely be significantly lower than the actual frequency of mutations in A/M.

**Figure 2:**
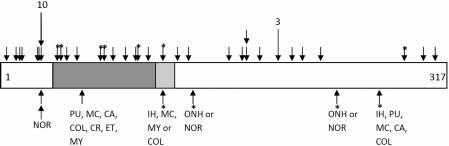
Schematic drawing of the SOX2 protein with mutations indicated. Mutations resulting in A/M phenotype (unilateral or bilateral) are shown on the top while mutations resulting in non-microphthalmia phenotypes (bilateral) are indicated below; missense mutations are indicated with asterisks. Each specific mutation is marked with a single arrow, unless the mutation resulted in both A/M and non-A/M ocular phenotypes, in which case the mutation is marked with an arrow above and below. Recurrent mutations are indicated with longer arrows with the number of families listed above. The dark gray box represents the homeodomain and the light gray box a partner-factor interaction domain. In the image, NOR indicates Normal; PU indicates Pupillary abnormality; MC indicates Microcornea; CA indicates Cataract; COL indicates Coloboma; CR indicates Chorioretinal dystrophy; ET indicates Esotropia; MY indicates High myopia; IH indicates Iris hypoplasia; ONH indicates Optic nerve hypoplasia.

Previously reported mutations in *SOX2* associated with A/M or non-A/M phenotypes are scattered throughout the gene with no particular pattern identified (Figure 2). No disease-causing mutations were identified in 61 patients with ocular anomalies other than anophthalmia/microphthalmia, including 20 with phenotypes which specifically match those seen in the family reported by Mihelec et al. [12]: iris hypoplasia, pupil anomalies, coloboma, and optic nerve hypoplasia. In all, 28 patients with anterior segment anomalies and an additional five patients with coloboma were screened with no disease-causing mutations identified. The *SOX2* sequence appears to be extremely conserved with only one synonymous polymorphism (c.453G>A) observed in our patient group (also seen in controls).

Our data suggests that *SOX2* mutations are not a common cause of ocular defects other than anophthalmia/microphthalmia. Further studies with larger numbers of different phenotypes may reveal additional mutations, but there is no evidence at this time to suggest that routine screening of *SOX2* in patients with other ocular phenotypes is warranted.
